# Uncovering the global ranking of greenhouse gases intensity, efficiency and structural transformation

**DOI:** 10.1038/s41598-023-45389-5

**Published:** 2023-10-23

**Authors:** Muhammad Saleem, Muhammad Aslam, Azhar Ali Janjua

**Affiliations:** 1 Department of Industrial Engineering, Faculty of Engineering-Rabigh, King Abdulaziz University, 21589 Jeddah, Saudi Arabia; 2 Department of Statistics, Faculty of Science, King Abdulaziz University, 21551 Jeddah, Saudi Arabia; 3 Deputy Director Colleges Hafizabad, Higher Education Department, Punjab, Pakistan, Hafizabad, 52110 Pakistan

**Keywords:** Climate sciences, Environmental sciences, Energy science and technology

## Abstract

This study incorporated the index decomposition analysis to drive the GHGs emissions intensity and separate the impact into true efficiency and structural transformation of economic activities on GHGs emissions. The global perspective is investigated in three aspects; (i) global groups of countries regarding income level, (ii) global countries bifurcated into geo-political regions and, (iii) hundred countries are taken to perform individual country level analysis, by taking 20 years (2000–2019) data. The hundred countries are ranked regarding GHGs intensity, efficiency and economic activities with respect to (i) for the latest year performance, (ii) twenty years average performance and, (iii) annual average reduction of GHGs emissions, comparable with base year 2000. Income-based countries groups explicate the improvement of GHGs intensity for HIC only and the economic transformation contributed to it. Efficiency index for all income groups while economic activities of LMIC, MIC and UMIC deteriorated the GHGs emissions. Global geo-political regions explicate the mixed pattern of GHGs intensity. Efficiency index is best for Azerbaijan and least for Uzbekistan. While ranking average GHGs emission reduction countries; Zimbabwe is the best and Qatar is the last country in the intensity list.

## Introduction

Anthropogenic activities mainly through emissions of greenhouse gases (GHGs) have witnessed global warming and surface temperature is realizing 1.1C^o^ beyond 1850–1900 in 2011–2020^1^. Globally, the emissions of GHGs [like, carbon dioxide (CO_2_), nitrous oxide (N_2_O), methane (CH_4_), hydrofluorocarbons (HFCs), sulphur hexafluoride (SF_6_) and perfluorocarbons (PFCs)] are rising unprecedented level because of energy use, change of land use, consumption and production pattern between and within countries. The Earth elapsed about 5000 years to raise temperature about 5 °C while in the coming century global temperature is expected to increase from 2 °C to 6 °C^2–4^. The impact of climate change hindering lives on earth through multiple facets and the literature is evident of its socio-economic aspects as well. Emissions gap report^5^ evident of inadequate measures to deal with global climate crises which required urgent social and economic transformations. United Nations Climate Change Conference of the Parties (COP 26) endorsed the slight progress in limiting the emission gap for 2030 to achieve the objective of the Paris Agreement to limit the increasing temperature whereas the prevailing policies led to a rise in global temperature by 2.8C^o^ at the end of this century. To limit the warming rate to the goal of the Paris Agreement (that is 1.5C^o^) GHGs emissions are required to be reduced by 45% through broad based, wide rage, large scale and systematic transformations. China, the USA, India and EU27 are the leading total GHGs emissions based countries while the USA, Russian Federation and China are the per capita GHGs emissions based countries, respectively.

The considerations related to climate change emerged from energy sources. Fuels are important sources to provide utmost world’s energy and they provide foundations for emissions of GHGs^6^ and increasing warming rate. Energy consumption is a significantly important factor for all sectors of the economy but its consumption is creating environmental complications by engulfing other spheres of life on the globe and necessitates serious warnings of threats to avoid its glitches^6^. Energy consumption is a key input factor during the production process with limited but varying levels of substitutability, globally and country to country^7^. By the year 2040, the global population is likely to increase from 7 to 9 billion which will increase the energy demand. Fundamental requirements of energy consumption and the emergence of its harmful environmental impact require the balancing state of sustainability which becomes a challenging task in developed and developing economies of the world^8–10^ where maladaptation and insufficient financial flow are hindering the implementations of adaptation measures^1^.

Table 1 explains the energy and emissions pattern in various global regions which clarifies the improvements in Europe, Eurasia and North America while disregarding the development in Asia Pacific and other regions.

**Table 1:** Percentage Change in Energy and CO_2_ Emission by 2021 (base 1990).

Region	Energy supply (Mtoe)	Elect. cons. (TWh)	CO_2_ emissions (Mt)	CO_2_/Capita (t)
World	65.79	129.67	63.92	13.14
Europe	− 6.89	25.97	26.25	− 32.72
Africa	122.82	155.63	140	15.1
Asia Pacific	184.76	435.29	238.19	138.55
Central and South America	89.66	154.82	91.81	32.92
Eurasia	− 11.16	2.71	− 22.61	− 29.57
Middle East	110.6	417.18	227.49	71.5
North America	20.12	45.67	4.86	-22.21

Source: IEA, 2021.

The rest of the study is structured as follows. Section “Literature review” illustrates the literature review, Section “Methodology” explains the methodology and describes the data, Section “Results and discussion”, reports the empirical results, interpretation with discussion, Section “Conclusion”, provides conclusion and policy implications. In Sect. 6, the limitations of the study for future research are given followed by references.

## Literature review

Renewable energy has emerged as a forceful bounce to reduce GHGs emissions and developed nations are marching toward its sources with ever growing speed to cater to the needs of global energy^11^. Reference^12^ assessed the European Union’s desire to become the first climate neutral continent by 2050. Using decoupling transport related GHGs emissions and economic growth from 1997 to 2017. The countries achieved relatively 55% and absolutely 34%. However, just 8 countries achieved strong decoupling which seems insufficient to achieve the target. Meanwhile, developing countries are formulating policies, and executing projects to implement the framework for the provision of rural renewable energy^11,13^.

Reference^14^ inverstigated the reduction in carbon emissions as a result of the Paris agreement. However, the nexus of economic growth and environmental sustainability remained under discussion which is explored. The agreement reveals about 4.1% emission reduction however the performance of the countries differ due to their income level. Efficiency and technical progress witnessed in developed countries and to reach net zero carbon emission new approaches especially considering the low income countries should be addressed. Reference^15^ probed the relationship between climate vulnerability and green investment using the data of 107 countries from 1995 to 2019. Socio-economic climate vulnerability decreases the technological investment for climate change adaptation and mitigation, whereas it is prominent in countries with lower development, facing energy restrictions and lesser innovation. Keeping in view the compulsory transition, the energy markets are progressively alluring the stakeholders^16^ but require a handsome amount of investment^11,17,18^ which hinders developing nations. Alternatively, environmental challenges emerged from the utilization of inefficient energy sources creating unpredictable costs^19^ which seems non-reversible damages as well. Energy efficiency may not lessen the emissions if producers remain unable to use clean energy so, the association of circular economy may lead toward sustainability.

Reference^20^ analyzed the impact of financial development to abate the carbon emissions for Mexico, Indonesia, Nigeria and Turkey from 1969 to 2019. Mexico and Turkey revealed a one-way casual association. A significant positive association exists between financial development and carbon emissions and this study recommends effective monetary policy to encourage institutes to work for emission decreasing tendencies^21^. Investigated the role of energy efficiency with emissions of GHGs and studied the association of digitalization and environmental technological innovations of G7 economies from 1990 to 2020. Sustainability increased with energy efficiency through environmental technological innovations. Growth is a significant factor in enhancing GHGs emissions thus investment in sustainable innovation is suggested to be formulated for net zero emissions.

Reference^22^ found that the characteristics of carbon emission efficiency vary with stages of development and these characteristics are important for policy formulation. With the development low carbon emissions are expected and recommended to formulate regional emission reduction zoning. For low carbon emission four aspects, ecological preservation, land use optimization, low carbon development and innovation cooperation are recommended. Reference^23^ investigated the link between political ideology and GHGs emissions for 98 countries from 1990 to 2016. Left-wing governments exhibit more GHGs compared with right-wing governments. The energy efficiency increases the with level of education and leads to less emissions of GHGs.

A circular economy is a recreating mechanism in which emissions, waste and inputs are diminished by recycling, refurbishing, reusing and repurposing (United Nations Climate Change, 2019). It guarantees efficient utilization of resources along with the reduction in environmental damages (The Circularity Gap Report, 2019) and is an important tool to reduce the production of GHGs which is disregarded in accomplishing the Paris Agreement. Efficient energy utilization seems to ascertain a desirable future environment which may be realized with effectively adopting the strategy of circular economy^24^.

Reference^25^ studied the urban–rural sources of GHGs for policy intervention regarding rapid urbanization. Rural emerging regions emit the majority of GHGs primarily from food and housing while in high income regions, significant GHGs are driven from transport and services. Reference^12^ studied the transport related GHGs emissions and growth in European Union countries using the decoupling method and found just 8 countries have achieved the desired decoupling. Reference^26^ evaluated the impact of European Union energy policy in decoupling growth from emission of GHGs. It asserts that outsourcing the GHGs emissions by relocating energy intensive industries to developing countries and supports the Pollution Heaven Hypothesis (PHH) and suggests investigating consumption based accounting rather production based accounting. Reference^27^ suggested treating the reduction of GHGs as a global public good so emphasized making it efficient with collective wisdom. The initiatives of rich countries will impact more for this cause. Labor and land costs are lower in low income countries and it is cheaper to build green than retrofit green so the global targets for the investment of development economies in low and middle income bears the significant importance to integrate the world.

Agriculture production doubled since 1970 by contributing 25% of global GHGs in 2010^28^. More food is possible with certain levels of GHGs so increasing food security by other means is required to be explored to curtail environmental degradation. Unsustainable urbanization is creating difficulties which is expected to double by 2050. The world is facing resource scarcity along with a rising population the catastrophe is emerging with environmental degradation which is a challenging task for the policy makers to ensure sustainability^29^. Reference^30^ probe the ten energy efficient countries to gauge the impact of energy efficiency on economic growth to mitigate environmental degradation and found that energy intensity has a positive impact on economic growth and degraded environment whereas energy efficiency bears the potential to reduce GHGs by about 40%.

The scarcity of oil and gas compels China to rely on coal which contributes 21% of global CO_2_^31,32^. Reference^33^ focused on air pollution and GHGs which are hindering sustainable growth in China^34^. Reference^35^ analyzed the determinants of climate change mitigation technology while focusing on the oil price as the cost of primary energy taking 30 countries from 1990 to 2019. The high energy intensive countries move toward renewable energy by investing in climate change mitigation technologies and lowering the emissions of GHGs. Reference^36^ studied the alternative energy sources for the shipping industry to reduce GHGs to contribute to the United Nations SDGs-2030. The study proposed biofuel may alternatively be used which potentially reduce marine transportation emission of GHGs from 25 to 100%. However, the supply of biofuels is insufficient and can meet 15% of the demand. Reference^37^ found that the ability of the managerial staff determined the emissions of GHGs. The problems of humans’ health and the degradation of the ecosystem are emerging while the economic priority makes it difficult to rapidly transform the economic structure and energy mix in short run whereas the scenario is unsuitable for sustainable development.

The available literature asserts that economic growth is the primary source of GHGs emissions^38–40^ and the trade liberalization through composition effect has a critical role in influencing it^41,42^. Population growth demands more food, housing, transportation^12^, and energy demands^30^ along with other requirement which compel agriculture^28^, urbanization^25^ and other services to increase the output which emit GHGs emissions. The literature recommends; the usage of sustainable urbanization, research in the agriculture sector, usage of clean/renewable energy, efficient utilization of resources (circular economy), and the export of inherent GHGs emissions so the usage of clean technologies^43^ to replace the production process (technical effect) and other adaptation measures. Reference^44^ discussed the factors including energy resources which are influencing economic growth. Environment and economic growth nexus is leading to new international agreements for green economies. Renewable energy utilization is showing more positive economic and environmental impact in middle income countries than in high income countries. The macroeconomic indicators are suggested to be of significant importance for the policy formulation.

A good quantity of literature addresses CO_2_ emissions while focusing on GHGs emissions from energy consumption and uses index number decomposition to provide consistent statistic to gauge energy efficiency and/or activity indices for selected countries. This study broadened the scope and incorporated the country level GHGs emissions which remained an ultimate objective while addressing clean/renewable energy, pollution, green economy, environment, sustainability and other related issues. This investigation revisits the comparative status of global geographic groups of countries, groups of countries bifurcated with respect to income level and the individual country level analysis of a hundred selected countries. We incorporate brief GHGs emissions intensity decomposition to bifurcate the central developments in GHGs efficiency and structural shifts in economic activities that bring deviations in GHGs emissions intensities^33^. This study provides the ranking of a hundred countries according to; for the latest year, twenty years’ average and average annual GHGs emission reduction performance along with a ranking of major contributions (efficiency and activity).

## Methodology

Decoupling refers to the separation of two or more activities and here it means the growth without generating GHGs. Relative and absolute terms highlight the level of intensity of GHGs emissions with growth whereas both endure close association in the same direction. Improvements in the efficiency of GHGs emissions are the most cost effective and readily available alternative to support sustainability^45^. To achieve the UN-SGD of sustainability it is pertinent to understand how the economies may grow with low emission future. GHGs emissions level changes with variety of factors therefore, index number decomposition provides a reliable method to gauge GHGs intensity and determine of efficiency index or activity index while the decomposition methodology is useful for comprehending the pattern^46^ of GHGs emissions.

Numerous decoupling methodologies are evident in literature^24,32,40,45,47–52^ to successfully disseminate valuable dimensions that contribute to underlying factors to alter energy intensity. Decomposition analysis may be categorized in two ways; the first is structural decomposition analysis (SDA) and the second is index decomposition analysis (IDA).

Energy intensity can be assessed through an index approach using Fisher ideal index^24,45^, as given below:

1 $$EIt=\sqrt{Laspeyres \times Paache}\times 100$$

2 $$Laspeyres=\frac{\sum E1it \,Yoi}{\sum Eoi\, Yoi} \times 100$$

3 $$Paasche=\frac{\sum E1it \,Y1i}{\sum Eoi \,Y1i} \times 100$$

4 $$EFIt=\sqrt{Laspeyres \times Paache}$$

4.a $$ACIt=\sqrt{Laspeyres \times Paache}$$

Here *EI*_*t*_ denotes energy intensity in a specified period, *E* represents the consumption of energy and the output is represented with *Y*. Subscripts “*o ”, “1*”, “*t”* and “*I”* is used to denote the current year, base year, time and sector of the economy, respectively.

5 $$EIt=EFIt \times ACIt$$

6 $$EFt=\Delta ESt=\frac{EFIt}{EIt}+ \frac{ACIt}{EIt}$$

The intensity of GHGs, measures the average emissions of GHGs for the economic activities of a country and is stated as the ratio of total GHGs emissions per unit of GDP^7^. The decomposition extricates the reduction in GHGs emissions (efficiency index) because of structural change in economic activities. The index referred to GHGs emissions efficiency which shows GHGs emissions per unit of economic output.

### Data source

The latest, available twenty years (2000–2019) data was collected from World Development Indicators (WDI) in April 2023. Separation of countries regarding their economic, geographical and other details are also the same as described by WB-WDI. Data from the International Energy Agency (IEA) is also utilized and mentioned appropriately.

## Results and discussion

Table 2 shows the summary statistics of indices calculated for 100 countries. Index value with base year 2000 is presented followed by average value of the indices during twenty years and lastly, the summary statistics of indices for average reduction in GHGs during these twenty years are presented.

**Table 2:** Indices summary statistics of one hundred countries.

Indices		Obs	Mean	Std. Dev	Min	Max
Index value in 2019 (2000 = 1)	Efficiency	100	1.075	0.561	0.314	3.092
	Activity	100	1.088	0.524	0.297	3.299
	Intensity	100	1.037	0.498	0.354	2.839
Average values (2000–2019)	Efficiency	100	1.027	0.286	0.472	1.931
	Activity	100	1.056	0.284	0.667	2.262
	Intensity	100	1.030	0.258	0.649	2.168
Average annual change (%) 2000–2019	Efficiency	100	− 0.136	2.637	− 5.818	6.250
	Activity	100	0.026	2.394	− 5.743	6.529
	Intensity	100	− 0.268	2.419	− 5.167	5.877
Average GHG reduction 2000–2019	Efficiency	100	0.066	0.691	− 1.507	2.875
	Activity	100	− 0.038	0.611	− 2.522	1.297
	Intensity	100	0.028	0.518	− 1.510	0.996
	Countries	100	50.500	29.012	1	100

*Obs.* observations, *Std. Dev.* standard deviation, *Min.* minimum, *Max.* maximum.

Table 3 presents GHGs intensity index and decomposition outcomes of global income based groups of countries as bifurcated and endorsed by World Bank (WB) in World Development Indicators (WDI). Comparing with year 2000, with the exception of HIC the GHG intensity index has increased for rest of the groups which partially endorsed the results of Ref.^14^ because the study in hand is using the larger dataset and global groups formulated by WB. The group of HIC is 19 percent less GHG intensive and the economic transformation favored this change while inefficiency deteriorated it than in year 2000, showing the same direction as suggested by Ref.^12^ while the magnitude differs due to different time periods and countries. For the rest of the groups, GHG intensity index increased dominantly by the rise in the inefficiency index. The average indices from 2000 to 2019 confirm some positive fluctuations in the economic activity index with the exception of three groups (LMIC, MIC and UMIC). The average efficiency index in all groups and intensity index with the exception of HIC has deteriorated, endorsed the findings of Ref.^22^.

**Table 3:** Global GHGs intensity index and decomposition results of income based groups of countries.

Countries	Index value in 2019 (2000 = 1)			Average values (2000–2019)			Average annual change (%) (2000–2019)			Indices average GHG reduction (2000–2019)		
	Efficiency	Activity	Intensity	Efficiency	Activity	Intensity	Efficiency	Activity	Intensity	Efficiency	Activity	Intensity
HIPC	1.444	0.940	1.358	1.206	0.975	1.173	1.966	− 0.309	1.628	− 0.386	0.055	− 0.330
HIC	1.491	0.542	0.808	1.211	0.760	0.901	2.127	− 3.168	− 1.116	− 0.413	0.654	0.241
LDC	1.529	0.956	1.461	1.266	0.992	1.254	2.270	− 0.225	2.026	− 0.488	0.017	− 0.471
LMIC	1.548	1.058	1.638	1.224	1.084	1.330	2.334	0.309	2.635	− 0.410	− 0.169	− 0.580
LIC	1.608	0.783	1.259	1.272	0.909	1.139	2.555	− 1.257	1.233	− 0.498	0.219	− 0.278
LWMIC	1.559	0.979	1.525	1.279	0.987	1.262	2.367	− 0.107	2.252	− 0.509	0.029	− 0.480
MIC	1.541	1.070	1.649	1.219	1.092	1.335	2.307	0.372	2.672	− 0.401	− 0.185	− 0.586
UMIC	1.518	1.117	1.695	1.185	1.145	1.362	2.236	0.605	2.824	− 0.341	− 0.283	− 0.624

Where *HIPC* heavily indebted poor countries, *HIC* high income countries, *LDC* leased developed countries, *LMIC* low and middle income countries, *LIC* low income countries, *LWNIC* lower middle income countries, *MIC* middle income countries, *UMIC* upper middle income countries.

Table 4 explicates the GHGs intensity index and decomposition analysis of global geo-political regions based on available latest twenty years’ data from WDI. The region wise outcomes indicate wide variations in three indices. There are noteworthy comparative disparities in the transformation of economic activities and in the efficiencies across regions. Compared with the year 2000, in 2019 out of 14 global regions just 6 regions (Euro area, Europe and Central Asia, European Union, Latin America and the Caribbean, OECD members and, Central African Republic) are less GHG intensive. In the year 2019, the GHG intensity index of these six regions is declined by 22 percent whereas in the eight regions (Africa Eastern and Southern, Africa Western and Central, Arab World, Central Europe and the Baltics, East Asia and Pacific, Middle East and North Africa, South Asia and, Sub Saharan Africa) the intensity index increased by 31 percent. On average, the rise in GHG intensity is due to alterations in both GHG efficiency and economic activities. The finding endorsed the earlier studies of Refs.^12,21^ which state the progress based on the characteristics of the countries.

**Table 4:** Global GHGs intensity and decomposition indices of geo-political groups.

Countries	Index value in 2019 (2000 = 1)			Average values (2000–2019)			Average annual change (%) (2000–2019)			Indices average GHG reduction (2000–2019)		
	Efficiency	Activity	Intensity	Efficiency	Activity	Intensity	Efficiency	Activity	Intensity	Efficiency	Activity	Intensity
Africa Eastern and Southern	1.327	0.861	1.143	1.138	0.983	1.113	1.516	− 0.764	0.713	− 0.264	0.040	− 0.112
Africa Western and Central	1.774	0.836	1.484	1.511	0.885	1.324	3.126	− 0.913	2.128	− 0.883	0.276	− 0.476
Arab World	1.091	1.021	1.114	1.051	1.041	1.095	0.471	0.127	0.582	− 0.104	− 0.083	− 0.078
Central Europe and the Baltics	2.089	0.518	1.082	1.458	0.755	1.053	3.978	− 3.382	0.427	− 0.837	0.720	− 0.010
East Asia & Pacific	1.229	1.191	1.464	1.031	1.187	1.225	1.118	0.956	2.030	− 0.057	− 0.357	− 0.293
Euro area	1.586	0.456	0.723	1.216	0.727	0.854	2.475	− 4.031	− 1.688	− 0.431	0.809	0.460
Europe & Central Asia	1.470	0.545	0.801	1.201	0.769	0.902	2.059	− 3.135	− 1.157	− 0.394	0.634	0.328
European Union	1.609	0.465	0.749	1.230	0.730	0.867	2.553	− 3.932	− 1.508	− 0.456	0.796	0.423
Latin America & Caribbean	1.248	0.729	0.909	1.096	0.901	0.982	1.180	− 1.642	− 0.491	− 0.190	0.230	0.138
Middle East & North Africa	1.092	1.010	1.103	1.038	1.047	1.087	0.473	0.065	0.526	− 0.077	− 0.096	− 0.065
OECD members	1.517	0.528	0.801	1.221	0.751	0.895	2.223	− 3.300	− 1.158	− 0.434	0.690	0.344
South Asia	1.592	1.126	1.792	1.260	1.068	1.355	2.493	0.644	3.130	− 0.475	− 0.136	− 0.481
Sub-Saharan Africa	1.498	0.852	1.276	1.272	0.946	1.196	2.165	− 0.829	1.304	− 0.499	0.123	− 0.258
Central African Republic	1.488	0.455	0.677	1.328	0.671	0.878	3.617	− 3.553	− 1.479	− 0.626	0.967	0.424

All the regions indicate departure from GHG efficiency index while four regions (Arab World, East Asia and Pacific, Latin America and the Caribbean, and Middle East and North Africa) remained close to the level of base year (that is year 2000). Economic transformation toward GHG intensive economic and activities index is calculated in all regions which explains the improvements with the exception of four regions (Arab World, East Asia and Pacific, Middle East and North Africa, and South Asia) which demonstrate economic transformation toward inefficient economic activities lead to more GHG emission. In six (6) global regions (Euro Area, Europe and Central Asia, European Union, Latin America and Caribbean, OECD members and Central African Republic) improvements in GHG intensity are due to transformation toward less GHG intensive economic activities whereas, all of these regions show a rise in GHG inefficiencies reference to the base year. For example, in the Euro Area average GHG intensity index is 85% (an improvement of 15%): structural activities and GHG efficiency indices are 73% (favorable economic transformation impact is 27%) and 122% (inefficiency of 22%) of its base level, respectively. In all regions, economic structural shift is the dominant aspect in declining GHG intensity whereas in East Asia and the Pacific region, economic transformation deteriorates more relative to inefficiency. Compared with the year 2000, South Asia is the global leading GHG intensive region where economic transformation and efficiency indices both have deteriorated while the Euro Area followed by European Union and Central African Republic are the leading less GHG intensive regions, respectively. Investigation by regions illustrates the significant disparities in GHG intensity along with the directions of these deteriorations. Similarly, the groups of countries alienated with respect to income may provide thoughtful leadings for policy insinuation.

Table 5 elucidates the country level GHGs intensity and decomposition results of one hundred countries. Compared with the year 2000, the GHG intensity index of, 52 countries declined between 1 to 65%, 2 countries remained unchanged and the remaining 46 countries increased between 1 to 184% for the year 2019. In Denmark, Greece, Finland, Italy, Syrian Arab, Belgium, Germany, France, Netherlands, Hungary and Ireland (11 countries) GHG intensity reduced by more than 50% while this intensity increased above 50% in 13 countries whereas in Qatar, Turkmenistan, Tajikistan, Mongolia and Lao PDR (5 countries) GHG intensity increased above 100%, respectively. The activity index; declined up to 70% in 50 countries, unchanged for 2 countries and increased in 48 countries to 130%, from its level in year 2000. In Syrian Arab, Ukraine, U.K, Sweden, Yemen, Portugal, Spain and Romania (8 countries) this index reduced above by 50% while it increased above 50% in 16 countries whereas, in Myanmar, China, Azerbaijan, Cambodia and Lao PDR (5 countries) it raised more than 100%. The GHG efficiency index in the year 2019 improved in 54 countries up to 69%, remained unchanged in 6 countries and downgraded in 40 countries up to 201%, from the level of year 2000. In Myanmar, Azerbaijan, Ireland, Nigeria, Bulgaria, Cuba, Poland, Denmark and Cambodia (9 countries) GHG efficiency index improved above 50% while it downgraded by more than 50% in 19 countries whereas, Uzbekistan, Romania, Turkmenistan, Ukraine, Lithuania, Sweden, U.K and Singapore (8 countries) it downgraded above 100%. In 54 countries economic activity transformation dominated the efficiency index while in 37 countries both the activity index and efficiency index led to GHG improvements, in the year 2019 from a level in the year 2000. The analysis for the year varies rapidly with small perturbations of short runs while the average values may point out the pattern.

**Table 5:** Country level GHGs intensity index and decomposition results of hundred countries.

Countries	Index value in 2019 (2000 = 1)			Average values (2000–2019)			Average annual change (%) (2000–2019)			Indices average GHG reduction (2000–2019)		
	Efficiency	Activity	Intensity	Efficiency	Activity	Intensity	Efficiency	Activity	Intensity	Efficiency	Activity	Intensity
Algeria	0.944	1.048	0.990	0.925	1.074	0.993	− 0.277	0.265	− 0.021	0.166	− 0.151	0.016
Argentina	0.879	0.825	0.725	0.929	0.955	0.883	− 0.598	− 0.881	− 1.658	0.167	0.104	0.271
Australia	0.575	0.975	0.560	0.767	1.001	0.768	− 2.600	− 0.124	− 2.733	0.602	− 0.001	0.601
Austria	0.735	0.760	0.558	0.901	0.878	0.800	− 1.540	− 1.431	− 2.957	0.239	0.284	0.523
Azerbaijan	0.323	2.568	0.829	0.472	2.262	0.902	− 5.356	5.470	− 0.891	2.875	− 2.522	0.354
Bahrain	0.944	1.293	1.221	0.961	1.191	1.142	− 0.271	1.375	1.091	0.087	− 0.363	− 0.276
Bangladesh	0.605	1.764	1.068	0.813	1.300	1.030	− 2.595	3.041	0.363	0.491	− 0.557	− 0.066
Belgium	0.577	0.773	0.446	0.755	0.885	0.678	− 2.781	− 1.344	− 4.093	0.638	0.271	0.909
Bosnia	0.816	0.984	0.803	0.943	1.026	0.968	− 0.947	− 0.068	− 1.012	0.128	− 0.051	0.076
Brazil	0.898	0.876	0.786	0.946	0.995	0.941	− 0.542	− 0.666	− 1.223	0.120	0.015	0.134
Bulgaria	0.469	1.080	0.507	0.702	1.106	0.774	− 3.742	0.428	− 3.355	0.845	− 0.218	0.628
Cambodia	0.515	2.294	1.180	0.682	1.615	1.049	− 3.371	4.479	0.926	0.991	− 1.108	− 0.117
Cameroon	0.494	1.247	0.616	0.700	1.122	0.775	− 3.631	1.180	− 2.503	0.848	− 0.253	0.594
Canada	0.784	0.832	0.652	0.883	0.916	0.813	− 1.257	− 0.959	− 2.205	0.270	0.189	0.459
Chad	0.699	1.918	1.341	0.647	1.798	1.122	− 1.551	3.784	1.617	1.129	− 1.405	− 0.276
Chile	0.810	1.140	0.923	0.853	1.119	0.952	− 1.041	0.702	− 0.357	0.344	− 0.237	0.107
China	0.539	2.947	1.588	0.810	1.944	1.480	− 3.160	5.872	2.539	0.607	− 1.541	− 0.934
Colombia	0.656	1.169	0.766	0.788	1.102	0.860	− 2.177	0.832	− 1.369	0.540	− 0.207	0.333
Croatia	0.607	0.830	0.504	0.811	0.954	0.784	− 2.568	− 0.942	− 3.479	0.474	0.114	0.588
Cuba	0.471	1.138	0.536	0.655	1.154	0.743	− 3.841	0.717	− 3.192	1.026	− 0.318	0.708
Denmark	0.490	0.722	0.354	0.794	0.838	0.681	− 3.536	− 1.693	− 5.167	0.560	0.395	0.955
Dominican	0.608	1.430	0.869	0.800	1.155	0.905	− 2.550	1.925	− 0.663	0.527	− 0.304	0.224
Egypt	0.855	1.265	1.081	0.992	1.123	1.109	− 0.793	1.261	0.440	0.023	− 0.239	− 0.216
Finland	0.575	0.742	0.427	0.842	0.889	0.764	− 2.542	− 1.544	− 4.051	0.414	0.265	0.678
France	0.640	0.730	0.467	0.826	0.860	0.720	− 2.297	− 1.640	− 3.902	0.427	0.334	0.761
Germany	0.617	0.724	0.447	0.840	0.840	0.715	− 2.468	− 1.676	− 4.115	0.393	0.382	0.774
Ghana	0.686	1.778	1.219	0.800	1.340	1.049	− 1.875	3.111	1.162	0.515	− 0.616	− 0.101
Greece	0.658	0.578	0.380	0.823	0.847	0.707	− 2.110	− 2.758	− 4.920	0.429	0.421	0.851
Hungary	0.531	0.919	0.488	0.733	0.958	0.711	− 3.253	− 0.419	− 3.664	0.714	0.100	0.815
India	0.596	1.915	1.142	0.787	1.411	1.078	− 2.664	3.495	0.727	0.574	− 0.741	− 0.167
Indonesia	0.566	1.517	0.859	0.738	1.233	0.886	− 2.907	2.223	− 0.753	0.734	− 0.456	0.278
Iran	1.004	0.963	0.967	0.978	1.083	1.058	0.063	− 0.130	− 0.143	0.048	− 0.165	− 0.117
Iraq	1.008	1.130	1.139	0.998	0.939	0.930	0.491	1.750	0.987	0.009	0.170	0.179
Ireland	0.373	1.323	0.494	0.689	1.081	0.734	− 4.933	1.626	− 3.608	0.943	− 0.172	0.770
Israel	0.607	1.110	0.674	0.847	1.017	0.855	− 2.514	0.558	− 1.992	0.388	− 0.036	0.352
Italy	0.722	0.595	0.429	0.897	0.787	0.719	− 1.672	− 2.693	− 4.317	0.248	0.548	0.796
Japan	0.797	0.654	0.522	0.927	0.815	0.761	− 1.154	− 2.203	− 3.333	0.165	0.454	0.619
Jordan	1.301	1.000	1.301	1.217	1.028	1.253	1.465	0.060	1.426	− 0.409	− 0.058	− 0.467
Kazakhstan	0.549	1.821	1.000	0.782	1.560	1.191	− 2.856	3.251	0.282	0.621	− 0.994	− 0.373
Kenya	1.005	1.313	1.320	1.014	1.124	1.139	0.372	1.456	1.836	− 0.021	− 0.239	− 0.260
Korea, Rep	0.665	1.171	0.779	0.801	1.133	0.903	− 2.091	0.842	− 1.276	0.491	− 0.267	0.225
Kuwait	1.048	1.067	1.118	0.954	1.181	1.121	0.343	0.467	0.630	0.105	− 0.345	− 0.241
Kyrgyz	1.263	1.017	1.285	1.103	1.027	1.130	1.532	0.336	1.381	− 0.202	− 0.050	− 0.251
Lao PDR	1.009	2.100	2.119	1.168	1.314	1.492	0.413	4.430	4.043	− 0.303	− 0.515	− 0.818
Lebanon	0.971	1.063	1.032	1.128	1.019	1.144	0.057	0.578	0.263	− 0.245	− 0.030	− 0.275
Lithuania	2.225	0.550	1.224	1.562	0.799	1.188	4.340	− 3.032	1.165	− 0.993	0.604	− 0.389
Luxembourg	0.677	0.936	0.634	0.915	0.989	0.909	− 1.908	− 0.336	− 2.270	0.228	0.025	0.253
Madagascar	1.534	0.639	0.979	1.242	0.789	0.966	2.430	− 2.183	− 0.022	− 0.464	0.539	0.076
Malaysia	0.801	1.404	1.124	0.967	1.178	1.129	− 1.110	1.808	0.678	0.083	− 0.338	− 0.255
Mali	1.258	1.163	1.462	1.215	1.039	1.263	1.335	0.839	2.081	− 0.409	− 0.078	− 0.487
Mauritania	0.960	1.226	1.177	0.994	1.096	1.088	− 0.131	1.147	0.870	0.015	− 0.189	− 0.174
Mexico	0.863	0.816	0.704	0.994	0.891	0.887	− 0.749	− 1.057	− 1.815	0.017	0.246	0.263
Mongolia	1.972	1.075	2.120	1.649	0.910	1.514	3.801	0.553	4.094	− 1.080	0.221	− 0.859
Morocco	0.737	1.459	1.075	0.870	1.231	1.055	− 1.509	2.072	0.413	0.323	− 0.437	− 0.114
Mozambique	1.973	1.008	1.989	1.528	1.038	1.576	3.766	0.141	3.717	− 0.877	− 0.078	− 0.955
Myanmar	0.314	3.299	1.035	0.503	2.177	0.954	− 5.818	6.529	0.223	2.412	− 2.271	0.141
Nepal	1.379	0.943	1.301	1.195	0.915	1.092	1.760	− 0.245	1.422	− 0.373	0.193	− 0.180
Netherlands	0.641	0.745	0.477	0.832	0.860	0.724	− 2.262	− 1.534	− 3.779	0.410	0.331	0.741
New Zealand	1.575	0.615	0.969	1.252	0.811	0.991	2.438	− 2.503	− 0.154	− 0.478	0.501	0.022
Nigeria	0.461	1.619	0.746	0.591	1.468	0.829	− 3.817	2.636	− 1.446	1.400	− 0.919	0.481
Norway	1.394	0.550	0.766	1.158	0.780	0.888	1.809	− 3.069	− 1.388	− 0.314	0.584	0.270
Oman	0.683	1.485	1.014	0.798	1.276	1.003	− 1.889	2.212	0.134	0.512	− 0.516	− 0.004
Pakistan	0.839	1.264	1.061	0.919	1.132	1.038	− 0.900	1.254	0.342	0.181	− 0.259	− 0.078
Panama	1.453	1.193	1.734	1.222	1.096	1.343	2.252	1.150	2.972	− 0.393	− 0.190	− 0.583
Papua N.G	1.333	0.961	1.281	1.150	0.950	1.089	1.620	− 0.139	1.374	− 0.282	0.116	− 0.166
Paraguay	1.201	0.930	1.117	1.069	0.972	1.038	1.064	− 0.308	0.619	− 0.135	0.061	− 0.074
Peru	1.530	0.918	1.404	1.275	0.963	1.228	2.302	− 0.399	1.824	− 0.505	0.084	− 0.421
Philippines	0.609	1.584	0.964	0.755	1.220	0.902	− 2.555	2.457	− 0.148	0.653	− 0.423	0.229
Poland	0.489	1.161	0.568	0.743	1.060	0.780	− 3.651	0.802	− 2.905	0.707	− 0.127	0.580
Portugal	1.474	0.445	0.656	1.158	0.712	0.803	2.181	− 4.060	− 2.176	− 0.324	0.853	0.529
Qatar	1.646	1.724	2.839	1.436	1.451	2.168	2.784	2.993	5.877	− 0.747	− 0.764	− 1.510
Romania	2.540	0.478	1.214	1.678	0.729	1.135	5.100	− 3.745	1.086	− 1.201	0.899	− 0.303
Russian F	0.608	1.069	0.650	0.710	1.141	0.804	− 2.543	0.396	− 2.228	0.791	− 0.286	0.506
Saudi Arabia	1.120	1.061	1.188	1.102	1.056	1.166	0.648	0.364	0.950	− 0.201	− 0.112	− 0.313
Senegal	1.312	0.940	1.233	1.151	0.921	1.059	1.518	− 0.258	1.128	− 0.294	0.176	− 0.118
Singapore	2.032	0.700	1.421	1.609	0.787	1.238	3.880	− 1.812	1.902	− 1.045	0.568	− 0.477
South Africa	0.916	0.926	0.848	0.993	1.018	1.011	− 0.411	− 0.397	− 0.807	0.019	− 0.035	− 0.017
Spain	1.641	0.474	0.778	1.243	0.760	0.909	2.700	− 3.775	− 1.292	− 0.477	0.717	0.240
Sri Lanka	1.488	0.987	1.469	1.290	0.959	1.234	2.272	0.082	2.073	− 0.520	0.094	− 0.426
Sudan	1.002	0.777	0.779	1.126	0.903	1.020	0.195	− 1.276	− 1.160	− 0.243	0.221	− 0.022
Sweden	2.218	0.388	0.861	1.501	0.667	0.931	4.331	− 4.803	− 0.780	− 0.953	1.139	0.186
Switzerland	1.599	0.503	0.805	1.221	0.758	0.894	2.563	− 3.491	− 1.131	− 0.430	0.689	0.258
Syrian	1.455	0.297	0.432	1.345	0.682	0.880	2.164	− 5.743	− 3.785	− 0.710	1.297	0.587
Tajikistan	1.533	1.510	2.315	1.468	1.105	1.632	2.378	2.270	4.530	− 0.810	− 0.192	− 1.002
Tanzania	1.792	1.040	1.864	1.304	1.076	1.403	3.171	0.267	3.339	− 0.537	− 0.154	− 0.691
Thailand	1.236	0.960	1.187	1.113	1.019	1.133	1.130	− 0.192	0.922	− 0.221	− 0.039	− 0.261
Tunisia	1.273	0.790	1.006	1.152	0.916	1.050	1.296	− 1.202	0.053	− 0.296	0.193	− 0.103
Turkiye	1.395	0.985	1.375	1.194	0.976	1.168	1.847	− 0.003	1.750	− 0.367	0.053	− 0.313
Turkmenistan	2.495	1.012	2.525	1.527	1.072	1.632	5.237	0.273	5.059	− 0.830	− 0.146	− 0.976
Uganda	1.412	1.310	1.850	1.185	1.212	1.446	2.002	1.611	3.315	− 0.351	− 0.394	− 0.745
Ukraine	2.290	0.364	0.833	1.650	0.695	1.075	4.595	− 4.967	− 0.764	− 1.203	1.097	− 0.107
U.A.E	0.933	1.275	1.190	0.892	1.282	1.136	− 0.238	1.406	0.959	0.254	− 0.524	− 0.270
U.K	2.143	0.367	0.785	1.429	0.671	0.889	4.133	− 5.108	− 1.260	− 0.838	1.146	0.308
U.S.A	0.610	0.827	0.505	0.791	0.907	0.724	− 2.547	− 0.991	− 3.516	0.525	0.214	0.739
Uruguay	1.482	0.639	0.947	1.154	0.847	0.953	2.216	− 2.272	− 0.221	− 0.279	0.387	0.108
Uzbekistan	3.092	0.623	1.926	1.931	0.786	1.418	6.250	− 2.376	3.528	− 1.507	0.666	− 0.841
Vietnam	1.009	1.905	1.923	1.042	1.346	1.406	0.131	3.560	3.506	− 0.087	− 0.609	− 0.696
Yemen, Rep	1.310	0.391	0.513	1.108	0.851	0.907	1.771	− 3.813	− 3.067	− 0.232	0.568	0.337
Zambia	1.973	0.865	1.707	1.488	0.965	1.415	3.758	− 0.664	2.885	− 0.819	0.088	− 0.731
Zimbabwe	1.061	0.559	0.593	0.926	0.702	0.649	0.567	− 2.849	− 2.201	0.191	0.805	0.996

Twenty years (2000–2019) average GHG intensity index illustrates that 50 countries improved up to 35% while one country (Oman) remained unchanged whereas the remaining 49 countries distorted the intensity index up to 117%. In Zimbabwe, Belgium and Denmark (3 countries) it improved above 30% while fewer than 5% improvements are estimated in Algeria, New Zealand, Bosnia & Herzegovina, Madagascar, Myanmar, Uruguay and Chile (7 countries). Over twenty years; Qatar, Turkmenistan, Tajikistan, Mozambique, Mongolia, Lao PDR, China, Uganda, Uzbekistan, Zambia, Vietnam and Tanzania (12 countries) distorted in average GHG intensity index more than 40%. Twenty years’ average activity index of 47 countries improved upto 33% where Sweden, U.K, Syrian Arab, Ukraine, Zimbabwe, Portugal and Romania (7 countries) show more than 25% improvements, 52 countries distorted this economic transformation for GHG emissions where Azerbaijan, Myanmar, China, Chad, Cambodia, Kazakhstan, Nigeria, Qatar and India (9 countries) are showing more than 25% distortion, respectively. 57 countries are explaining an improved efficient index up to 53% while 43 countries are illustrating deteriorated efficient index up to 93%. Azerbaijan, Myanmar, Nigeria, Chad, Cuba, Cambodia and Ireland (7 countries) are the leading countries that improved their efficiency index by more than 30%, whereas, Uzbekistan, Romania, Ukraine, Mongolia, Singapore, Lithuania, Mozambique, Turkmenistan and Sweden (9 countries) are the least countries which deteriorate the efficiency index above 50%.

Twenty years (2000–2019) average GHG reduction from intensity index illustrates that 50 countries have intensified the GHG emissions, furthermore, Qatar, Tajikistan, Turkmenistan, Mozambique, China, Mongolia, Uzekistan, Lao PDR, Uganda, Zambia, Vietnam and Tanzania (13 countries) are the leading GHG intensified countries with more than 0.5 (kt = co2) rise in GHG emission whereas 23 countries have reduced GHG emission above 0.5 and Zimbabwe, Denmark, Belgium, Greece, Hungary, Italy, Germany, Ireland, France, Netherlands, USA and Cuba (12 countries) reduced GHG emission above 0.71 (kt = co2). On the basis of twenty years (2000–2019) average of 53 countries increased while 47 countries reduced GHG emissions. Economic transformation bears the leading role in GHG emission in Azerbaijan, Myanmar, China, Chad and Cambodia (5 countries) where more than 1.11 (kt) GHG increased while in Syrian Arab, UK, Sweden and Ukraine (4 countries) are the leading GHG reducing countries above 1.01 (kt) to 1.30 (kt). Efficiency level increased GHG emission in 43 countries while in 57 countries efficiency led to reduced GHG emissions. More than 1.03 (kt) GHG curtailed in Azerbaijan, Myanmar, Nigeria, Chad and Cuba (5 countries) which are the leading GHG reducing countries that emerged from efficiency while Uzebkistan, Ukraine, Romania, Mongolia and Singapore are the leading GHG emission countries where it increased more than 1 (kt) based on twenty years’ annual average. In 56 countries efficiency level while in 44 countries economic transformation is a playing leading role in the reduction of GHG emissions, aligning with earlier findings of Refs.^14,21^.

Supplementary Table S1 shows the ranking of countries regarding GHGs emissions efficiency during the process of generating their GDP. The ranking of the countries illustrates best to worst countries as all countries did not improve. The magnitude for the year 2019 is comparable with the year 2000. Supplementary Table S2 illustrates the ranking of 100 countries regarding changes in GHGs emissions due to structural or economic activities shift for the year 2019 comparable with the base year (that is 2000). The countries are ranked from best to worst however magnitude can be verified from Table 5 which shows that not all the countries improve their economic activities regarding GHGs emissions. Supplementary Table S3 explains the GHGs emission intensity index ranking from best to worst countries for the year the 2019 with reference to year 2000. According to it, Denmark’s GHGs emissions are the ideal while Qatar is the lowest country in this ranking.

Supplementary Table S4 shows the twenty years (2000–2019) average GHGs efficiency index and Azerbaijan ranked first while Uzebkistan stood last. The countries are ranked top to least. Supplementary Table S5 is illustrating the twenty years (2000–2019) average GHG activities index from best to worst. Sweden is ranked at the top of the list where economic transformation led to the discharge less GHGs and Azerbaijan ranked at the bottom of the list. Supplementary Table S6 ranks the 100 hundred countries based on twenty years (2000–2019) average GHGs intensity by taking the year 2000 as a reference. The countries are ranked from best performing to least. Zimbabwe ranked top performing while Qatar is the least performing country here.

Supplementary Table S7 demonstrates the annual average (2000–2019) GHG reduction from efficiency and ranked from best to worst countries. Azerbaijan is the top GHG reducing from efficiency and Uzbekistan ranked last in this ranking. Supplementary Table S8 explicates the countries’ twenty years’ annual average ranking regarding GHG reduction from economic activities. Syrian is the leading country while Azerbaijan is the least country in this ranking. Supplementary Table S9 ranks the countries based on GHGs emission reduction from intensity. The twenty years’ annual average GHGs reduction ranking shows Zimbabwe reduced the most GHGs and Qatar is bottom of the ranking.

## Conclusion

This study investigated the global groups of countries separated regarding income level, global geo-political regions of countries and individual country level analysis of a hundred countries. GHGs emissions intensity has been decomposed into GHGs efficiency and structural transformation of economic activities those bring deviations in GHGs emissions based on the latest available twenty years (2000–2019) data from WDI. This study enlisted the GHGs intensity, efficiency and economic activities transformation rankings of a hundred countries according to; (i) for the latest year, (ii) twenty years’ average performance and, (iii) annual average reduction of GHGs emissions, comparable with base year 2000. Income based countries groups explain the improvement of GHGs intensity for HIC only and the economic activities transformation contributed to it. The efficiency index for all income groups deteriorated the GHGs emissions along with the economic activities of LMIC, MIC and UMIC. Global geo-political regions illustrate the mixed pattern of GHGs intensity. Out of 14 regions 6 (Euro Area, Europe and Central Asia, European Union, Latin America and the Caribbean, OECD members and the Central African Republic) have improved GHGs intensity along with these regions economic transformation is contributing positively in the other 4 regions (African Eastern and Southern, Africa Western and Central, Central Europe and the Baltics, and Sub-Saharan Africa) as well. Euro Area and European Union are the leading GHGs reducing groups and transformation in economic activities is the leading role for it. In groups/regions, the efficiency level is deteriorating the GHGs emissions while economic transformation is giving a mixed pattern. The country level analysis conveys a clear pattern for policy intervention in the country. For the year 2019, Denmark is the best country which improve GHGs emissions intensity while Qatar is the least country, Syrian is the best activities transformed country and Myanmar is ranked the least country, while regarding efficiency, Myanmar is leading and Uzbekistan is the least efficient country in this list. Twenty years’ average pattern among 100 countries when comparing with reference year illustrates those GHGs emission intensity is the best for Zimbabwe and least for Qatar, Activities index is best for Sweden and least for Azerbaijan. The efficiency index is best for Azerbaijan and least for Uzbekistan. Zimbabwe is leading and Qatar is the least country from intensity, Syrian is leading and Azerbaijan is the least from activities transformation, Azerbaijan is ranked first while Uzbekistan is the last from efficiency improved for average GHGs emission reduction countries.

### Policy implications

Efficiency level is altering the GHGs emissions improvement so suggesting easing the process and conditions of green technology transfer. Income based groups of countries provide an assessment of expected alternation in GHGs emissions with respect to changes in income of the countries. The analysis also clarifies the directions and specific policies for the income based position of the countries. In leading countries, economic transformation is the major contributing aspect of reducing GHGs whereas Pollution Heaven Hypothesis (PHH) seems applicable so the enumeration of production based and consumption based GHGs emissions is suggested to clarify the global GHGs contribution. Geo-political regions should be addressed to re-enforce the outcome of the environmental policy directions and comparative analysis gives an opportunity to readdress the issue with more clarity. Individual country level policy should be determined. Countries focusing on one direction required to explore both dimensions to devise more environment friendly policies. The literature suggests the use of monetary policy to encourage the investment for sustainable technology and considering the reduction of GHGs as global public goods may positively impact the objective.

### Limitation

The countries achieving the emissions targets are relatively wealthy and expectedly, managed to replace their production process with low emissions therefore, the Pollution Heaven Hypothesis (PHH) is very important and may be incorporated in future studies. Production based and consumption based decoupling is a critical aspect of estimation and is significant for global policy insinuation which requires attention from future researchers.

### Supplementary Information

Supplementary Tables.

## Data Availability

Dataset is available on https://databank.worldbank.org/reports.aspx?source=world-development-indicators and may also be obtained from Azhar Ali Janjua (azharjanjua12@gmail.com).
