# Supplementary material for: Uncovering the global ranking of greenhouse gases intensity, efficiency and structural transformation
Source: Sci Rep. 2023 Oct 23;13:18040. doi: 10.1038/s41598-023-45389-5 (PMC10593789; doi:10.1038/s41598-023-45389-5)
Supplement: Supplementary file 1 — Supplementary Tables. [file 41598_2023_45389_MOESM1_ESM.docx]

**Supplementary Tables**

Table S1: Ranking of countries regarding GHGs efficiency index in year 2019 (2000=1)

| Ranking | Countries | Ranking | Countries | Ranking | Countries | Ranking | Countries |
| --- | --- | --- | --- | --- | --- | --- | --- |
| 1 | Myanmar | 26 | Germany | 51 | Algeria | 76 | Uganda |
| 2 | Azerbaijan | 27 | France | 52 | Bahrain | 77 | Panama |
| 3 | Ireland | 28 | Netherlands | 53 | Mauritania | 78 | Syrian A. R. |
| 4 | Nigeria | 29 | Colombia | 54 | Lebanon | 79 | Portugal |
| 5 | Bulgaria | 30 | Greece | 55 | Sudan | 80 | Uruguay |
| 6 | Cuba | 31 | Korea, Rep. | 56 | Iran I.R. | 81 | Sri Lanka |
| 7 | Poland | 32 | Luxembourg | 57 | Kenya | 82 | Peru |
| 8 | Denmark | 33 | Oman | 58 | Iraq | 83 | Tajikistan |
| 9 | Cameroon | 34 | Ghana | 59 | Vietnam | 84 | Madagascar |
| 10 | Cambodia | 35 | Chad | 60 | Lao PDR | 85 | New Zealand |
| 11 | Hungary | 36 | Italy | 61 | Kuwait | 86 | Switzerland |
| 12 | China | 37 | Austria | 62 | Zimbabwe | 87 | Spain |
| 13 | Kazakhstan | 38 | Morocco | 63 | Saudi Arabia | 88 | Qatar |
| 14 | Indonesia | 39 | Canada | 64 | Paraguay | 89 | Tanzania |
| 15 | Australia | 40 | Japan | 65 | Thailand | 90 | Mongolia |
| 16 | Finland | 41 | Malaysia | 66 | Mali | 91 | Zambia |
| 17 | Belgium | 42 | Chile | 67 | Kyrgyz R. | 92 | Mozambique |
| 18 | India | 43 | Bosnia H. | 68 | Tunisia | 93 | Singapore |
| 19 | Bangladesh | 44 | Pakistan | 69 | Jordan | 94 | United K. |
| 20 | Israel | 45 | Egypt A. R. | 70 | Yemen, Rep. | 95 | Sweden |
| 21 | Croatia | 46 | Mexico | 71 | Senegal | 96 | Lithuania |
| 22 | Russian F. | 47 | Argentina | 72 | Papua N. G. | 97 | Ukraine |
| 23 | Domin, R. | 48 | Brazil | 73 | Nepal | 98 | Turkmenistan |
| 24 | Philippines | 49 | South Africa | 74 | Norway | 99 | Romania |
| 25 | United S. | 50 | United A. E. | 75 | Turkiye | 100 | Uzbekistan |

Table S2: Ranking of countries regarding GHGs activities index in year 2019 (2000=1)

| Ranking | Countries | Ranking | Countries | Ranking | Countries | Ranking | Countries |
| --- | --- | --- | --- | --- | --- | --- | --- |
| 1 | Syrian A.R. | 26 | Austria | 51 | Jordan | 76 | United A.E. |
| 2 | Ukraine | 27 | Belgium | 52 | Mozambique | 77 | Bahrain |
| 3 | United K. | 28 | Sudan | 53 | Turkmenistan | 78 | Uganda |
| 4 | Sweden | 29 | Tunisia | 54 | Kyrgyz R. | 79 | Kenya |
| 5 | Yemen, R. | 30 | Mexico | 55 | Tanzania | 80 | Ireland |
| 6 | Portugal | 31 | Argentina | 56 | Algeria | 81 | Malaysia |
| 7 | Spain | 32 | U.S. | 57 | Saudi Arabia | 82 | Domin.R. |
| 8 | Romania | 33 | Croatia | 58 | Lebanon | 83 | Morocco |
| 9 | Switzerland | 34 | Canada | 59 | Kuwait | 84 | Oman |
| 10 | Norway | 35 | Zambia | 60 | Russian F. | 85 | Tajikistan |
| 11 | Lithuania | 36 | Brazil | 61 | Mongolia | 86 | Indonesia |
| 12 | Zimbabwe | 37 | Peru | 62 | Bulgaria | 87 | Philippines |
| 13 | Greece | 38 | Hungary | 63 | Israel | 88 | Nigeria |
| 14 | Italy | 39 | South Africa | 64 | Iraq | 89 | Qatar |
| 15 | New Zealand | 40 | Paraguay | 65 | Cuba | 90 | Bangladesh |
| 16 | Uzbekistan | 41 | Luxembourg | 66 | Chile | 91 | Ghana |
| 17 | Madagascar | 42 | Senegal | 67 | Poland | 92 | Kazakhstan |
| 18 | Uruguay | 43 | Nepal | 68 | Mali | 93 | Vietnam |
| 19 | Japan | 44 | Thailand | 69 | Colombia | 94 | India |
| 20 | Singapore | 45 | Papua N.G. | 70 | Korea, R. | 95 | Chad |
| 21 | Denmark | 46 | Iran, I.R. | 71 | Panama | 96 | Lao PDR |
| 22 | Germany | 47 | Australia | 72 | Mauritania | 97 | Cambodia |
| 23 | France | 48 | Bosnia H. | 73 | Cameroon | 98 | Azerbaijan |
| 24 | Finland | 49 | Turkiye | 74 | Pakistan | 99 | China |
| 25 | Netherlands | 50 | Sri Lanka | 75 | Egypt, A. R. | 100 | Myanmar |

Table S3: Ranking of countries regarding GHGs intensity index in year 2019 (2000=1)

| Ranking | Countries | Ranking | Countries | Ranking | Countries | Ranking | Countries |
| --- | --- | --- | --- | --- | --- | --- | --- |
| 1 | Denmark | 26 | Portugal | 51 | Madagascar | 76 | Senegal |
| 2 | Greece | 27 | Israel | 52 | Algeria | 77 | Papua N.G. |
| 3 | Finland | 28 | Mexico | 53 | Kazakhstan | 78 | Kyrgyz R. |
| 4 | Italy | 29 | Argentina | 54 | Tunisia | 79 | Nepal |
| 5 | Syrian A.R. | 30 | Nigeria | 55 | Oman | 80 | Jordan |
| 6 | Belgium | 31 | Colombia | 56 | Lebanon | 81 | Kenya |
| 7 | Germany | 32 | Norway | 57 | Myanmar | 82 | Chad |
| 8 | France | 33 | Spain | 58 | Pakistan | 83 | Turkiye |
| 9 | Netherlands | 34 | Korea, R. | 59 | Bangladesh | 84 | Peru |
| 10 | Hungary | 35 | Sudan | 60 | Morocco | 85 | Singapore |
| 11 | Ireland | 36 | United K. | 61 | Egypt, A. R. | 86 | Mali |
| 12 | Croatia | 37 | Brazil | 62 | Paraguay | 87 | Sri Lanka |
| 13 | U.S. | 38 | Bosnia H. | 63 | Kuwait | 88 | China |
| 14 | Bulgaria | 39 | Switzerland | 64 | Malaysia | 89 | Zambia |
| 15 | Yemen, R. | 40 | Azerbaijan | 65 | Iraq | 90 | Panama |
| 16 | Japan | 41 | Ukraine | 66 | India | 91 | Uganda |
| 17 | Cuba | 42 | South Africa | 67 | Mauritania | 92 | Tanzania |
| 18 | Austria | 43 | Indonesia | 68 | Cambodia | 93 | Vietnam |
| 19 | Australia | 44 | Sweden | 69 | Thailand | 94 | Uzbekistan |
| 20 | Poland | 45 | Domin.R. | 70 | Saudi Arabia | 95 | Mozambique |
| 21 | Zimbabwe | 46 | Chile | 71 | United A.E. | 96 | Lao PDR |
| 22 | Cameroon | 47 | Uruguay | 72 | Romania | 97 | Mongolia |
| 23 | Luxembourg | 48 | Philippines | 73 | Ghana | 98 | Tajikistan |
| 24 | Russian F. | 49 | Iran, I.R. | 74 | Bahrain | 99 | Turkmenistan |
| 25 | Canada | 50 | New Zealand | 75 | Lithuania | 100 | Qatar |

Table S4: Ranking of countries regarding GHGs efficiency index average 2000-2019 (2000=1)

| Ranking | Countries | Ranking | Countries | Ranking | Countries | Ranking | Countries |
| --- | --- | --- | --- | --- | --- | --- | --- |
| 1 | Azerbaijan | 26 | China | 51 | Malaysia | 76 | Nepal |
| 2 | Myanmar | 27 | Croatia | 52 | Iran, I.R. | 77 | Mali |
| 3 | Nigeria | 28 | Bangladesh | 53 | Egypt, A. R. | 78 | Jordan |
| 4 | Chad | 29 | Greece | 54 | South Africa | 79 | Switzerland |
| 5 | Cuba | 30 | France | 55 | Mexico | 80 | Panama |
| 6 | Cambodia | 31 | Netherlands | 56 | Mauritania | 81 | Madagascar |
| 7 | Ireland | 32 | Germany | 57 | Iraq | 82 | Spain |
| 8 | Cameroon | 33 | Finland | 58 | Kenya | 83 | New Zealand |
| 9 | Bulgaria | 34 | Israel | 59 | Vietnam | 84 | Peru |
| 10 | Russian F. | 35 | Chile | 60 | Paraguay | 85 | Sri Lanka |
| 11 | Hungary | 36 | Morocco | 61 | Saudi Arabia | 86 | Tanzania |
| 12 | Indonesia | 37 | Canada | 62 | Kyrgyz R. | 87 | Syrian A.R. |
| 13 | Poland | 38 | United A.E. | 63 | Yemen, R. | 88 | United K. |
| 14 | Belgium | 39 | Italy | 64 | Thailand | 89 | Qatar |
| 15 | Philippines | 40 | Austria | 65 | Sudan | 90 | Tajikistan |
| 16 | Australia | 41 | Luxembourg | 66 | Lebanon | 91 | Zambia |
| 17 | Kazakhstan | 42 | Pakistan | 67 | Papua N.G. | 92 | Sweden |
| 18 | India | 43 | Algeria | 68 | Senegal | 93 | Turkmenistan |
| 19 | Colombia | 44 | Zimbabwe | 69 | Tunisia | 94 | Mozambique |
| 20 | U.S. | 45 | Japan | 70 | Uruguay | 95 | Lithuania |
| 21 | Denmark | 46 | Argentina | 71 | Norway | 96 | Singapore |
| 22 | Oman | 47 | Bosnia H. | 72 | Portugal | 97 | Mongolia |
| 23 | Ghana | 48 | Brazil | 73 | Lao PDR | 98 | Ukraine |
| 24 | Domin.R. | 49 | Kuwait | 74 | Uganda | 99 | Romania |
| 25 | Korea, R. | 50 | Bahrain | 75 | Turkiye | 100 | Uzbekistan |

Table S5: Ranking of countries regarding GHGs activities index average 2000-2019 (2000=1)

| Ranking | Countries | Ranking | Countries | Ranking | Countries | Ranking | Countries |
| --- | --- | --- | --- | --- | --- | --- | --- |
| 1 | Sweden | 26 | Belgium | 51 | Lebanon | 76 | Russian F. |
| 2 | United K. | 27 | Finland | 52 | Thailand | 77 | Cuba |
| 3 | Syrian A.R. | 28 | Mexico | 53 | Bosnia H. | 78 | Domin.R. |
| 4 | Ukraine | 29 | Sudan | 54 | Kyrgyz R. | 79 | Malaysia |
| 5 | Zimbabwe | 30 | U.S. | 55 | Jordan | 80 | Kuwait |
| 6 | Portugal | 31 | Mongolia | 56 | Mozambique | 81 | Bahrain |
| 7 | Romania | 32 | Nepal | 57 | Mali | 82 | Uganda |
| 8 | Switzerland | 33 | Tunisia | 58 | Saudi Arabia | 83 | Philippines |
| 9 | Spain | 34 | Canada | 59 | Poland | 84 | Morocco |
| 10 | Norway | 35 | Senegal | 60 | Turkmenistan | 85 | Indonesia |
| 11 | Uzbekistan | 36 | Iraq | 61 | Algeria | 86 | Oman |
| 12 | Singapore | 37 | Papua N.G. | 62 | Tanzania | 87 | United A.E. |
| 13 | Italy | 38 | Croatia | 63 | Ireland | 88 | Bangladesh |
| 14 | Madagascar | 39 | Argentina | 64 | Iran, I.R. | 89 | Lao PDR |
| 15 | Lithuania | 40 | Hungary | 65 | Panama | 90 | Ghana |
| 16 | New Zealand | 41 | Sri Lanka | 66 | Mauritania | 91 | Vietnam |
| 17 | Japan | 42 | Peru | 67 | Colombia | 92 | India |
| 18 | Denmark | 43 | Zambia | 68 | Tajikistan | 93 | Qatar |
| 19 | Germany | 44 | Paraguay | 69 | Bulgaria | 94 | Nigeria |
| 20 | Uruguay | 45 | Turkiye | 70 | Chile | 95 | Kazakhstan |
| 21 | Greece | 46 | Luxembourg | 71 | Cameroon | 96 | Cambodia |
| 22 | Yemen, R. | 47 | Brazil | 72 | Egypt, A. R. | 97 | Chad |
| 23 | France | 48 | Australia | 73 | Kenya | 98 | China |
| 24 | Netherlands | 49 | Israel | 74 | Pakistan | 99 | Myanmar |
| 25 | Austria | 50 | South Africa | 75 | Korea, R. | 100 | Azerbaijan |

Table S6: Ranking of countries regarding GHGs intensity index average 2000-2019 (2000=1)

| Ranking | Countries | Ranking | Countries | Ranking | Countries | Ranking | Countries |
| --- | --- | --- | --- | --- | --- | --- | --- |
| 1 | Zimbabwe | 26 | Colombia | 51 | Oman | 76 | Kenya |
| 2 | Belgium | 27 | Syrian A.R. | 52 | South Africa | 77 | Bahrain |
| 3 | Denmark | 28 | Argentina | 53 | Sudan | 78 | Lebanon |
| 4 | Greece | 29 | Indonesia | 54 | Bangladesh | 79 | Saudi Arabia |
| 5 | Hungary | 30 | Mexico | 55 | Pakistan | 80 | Turkiye |
| 6 | Germany | 31 | Norway | 56 | Paraguay | 81 | Lithuania |
| 7 | Italy | 32 | United K. | 57 | Ghana | 82 | Kazakhstan |
| 8 | France | 33 | Switzerland | 58 | Cambodia | 83 | Peru |
| 9 | Netherlands | 34 | Azerbaijan | 59 | Tunisia | 84 | Sri Lanka |
| 10 | U.S. | 35 | Philippines | 60 | Morocco | 85 | Singapore |
| 11 | Ireland | 36 | Korea, R. | 61 | Iran, I.R. | 86 | Jordan |
| 12 | Cuba | 37 | Domin.R. | 62 | Senegal | 87 | Mali |
| 13 | Japan | 38 | Yemen, R. | 63 | Ukraine | 88 | Panama |
| 14 | Finland | 39 | Luxembourg | 64 | India | 89 | Tanzania |
| 15 | Australia | 40 | Spain | 65 | Mauritania | 90 | Vietnam |
| 16 | Bulgaria | 41 | Iraq | 66 | Papua N.G. | 91 | Zambia |
| 17 | Cameroon | 42 | Sweden | 67 | Nepal | 92 | Uzbekistan |
| 18 | Poland | 43 | Brazil | 68 | Egypt, A. R. | 93 | Uganda |
| 19 | Croatia | 44 | Chile | 69 | Kuwait | 94 | China |
| 20 | Austria | 45 | Uruguay | 70 | Chad | 95 | Lao PDR |
| 21 | Portugal | 46 | Myanmar | 71 | Malaysia | 96 | Mongolia |
| 22 | Russian F. | 47 | Madagascar | 72 | Kyrgyz R. | 97 | Mozambique |
| 23 | Canada | 48 | Bosnia H. | 73 | Thailand | 98 | Tajikistan |
| 24 | Nigeria | 49 | New Zealand | 74 | Romania | 99 | Turkmenistan |
| 25 | Israel | 50 | Algeria | 75 | United A.E. | 100 | Qatar |

Table S7: Ranking of countries regarding GHGs reduction with efficiency average 2000-2019

| Ranking | | Countries | Ranking | | Countries | Ranking | | Countries | Ranking | | Countries | |
| --- | --- | --- | --- | --- | --- | --- | --- | --- | --- | --- | --- | --- |
| 1 | Azerbaijan | | 26 | Korea, R. | | 51 | Malaysia | 76 | Nepal | |  |  |
| 2 | Myanmar | | 27 | Bangladesh | | 52 | Iran, I.R. | 77 | Panama | |  |  |
| 3 | Nigeria | | 28 | Croatia | | 53 | Egypt, A. R. | 78 | Jordan | |  |  |
| 4 | Chad | | 29 | Greece | | 54 | South Africa | 79 | Mali | |  |  |
| 5 | Cuba | | 30 | France | | 55 | Mexico | 80 | Switzerland | |  |  |
| 6 | Cambodia | | 31 | Finland | | 56 | Mauritania | 81 | Madagascar | |  |  |
| 7 | Ireland | | 32 | Netherlands | | 57 | Iraq | 82 | Spain | |  |  |
| 8 | Cameroon | | 33 | Germany | | 58 | Kenya | 83 | New Zealand | |  |  |
| 9 | Bulgaria | | 34 | Israel | | 59 | Vietnam | 84 | Peru | |  |  |
| 10 | Russian F. | | 35 | Chile | | 60 | Paraguay | 85 | Sri Lanka | |  |  |
| 11 | Indonesia | | 36 | Morocco | | 61 | Saudi Arabia | 86 | Tanzania | |  |  |
| 12 | Hungary | | 37 | Canada | | 62 | Kyrgyz R. | 87 | Syrian A.R. | |  |  |
| 13 | Poland | | 38 | United A.E. | | 63 | Thailand | 88 | Qatar | |  |  |
| 14 | Philippines | | 39 | Italy | | 64 | Yemen, R. | 89 | Tajikistan | |  |  |
| 15 | Belgium | | 40 | Austria | | 65 | Sudan | 90 | Zambia | |  |  |
| 16 | Kazakhstan | | 41 | Luxembourg | | 66 | Lebanon | 91 | Turkmenistan | |  |  |
| 17 | China | | 42 | Zimbabwe | | 67 | Uruguay | 92 | United K. | |  |  |
| 18 | Australia | | 43 | Pakistan | | 68 | Papua N.G. | 93 | Mozambique | |  |  |
| 19 | India | | 44 | Argentina | | 69 | Senegal | 94 | Sweden | |  |  |
| 20 | Denmark | | 45 | Algeria | | 70 | Tunisia | 95 | Lithuania | |  |  |
| 21 | Colombia | | 46 | Japan | | 71 | Lao PDR | 96 | Singapore | |  |  |
| 22 | Domin.R. | | 47 | Bosnia H. | | 72 | Norway | 97 | Mongolia | |  |  |
| 23 | U.S. | | 48 | Brazil | | 73 | Portugal | 98 | Romania | |  |  |
| 24 | Ghana | | 49 | Kuwait | | 74 | Uganda | 99 | Ukraine | |  |  |
| 25 | Oman | | 50 | Bahrain | | 75 | Turkiye | 100 | Uzbekistan | |  |  |

Table S8: Ranking of countries regarding GHGs reduction with activities average 2000-2019

| Ranking | Countries | Ranking | Countries | Ranking | Countries | Ranking | Countries |
| --- | --- | --- | --- | --- | --- | --- | --- |
| 1 | Syrian A.R. | 26 | Belgium | 51 | Israel | 76 | Russian F. |
| 2 | United K. | 27 | Finland | 52 | Thailand | 77 | Domin.R. |
| 3 | Sweden | 28 | Mexico | 53 | Kyrgyz R. | 78 | Cuba |
| 4 | Ukraine | 29 | Sudan | 54 | Bosnia H. | 79 | Malaysia |
| 5 | Romania | 30 | Mongolia | 55 | Jordan | 80 | Kuwait |
| 6 | Portugal | 31 | U.S. | 56 | Mali | 81 | Bahrain |
| 7 | Zimbabwe | 32 | Nepal | 57 | Mozambique | 82 | Uganda |
| 8 | Spain | 33 | Tunisia | 58 | Saudi Arabia | 83 | Philippines |
| 9 | Switzerland | 34 | Canada | 59 | Poland | 84 | Morocco |
| 10 | Uzbekistan | 35 | Senegal | 60 | Turkmenistan | 85 | Indonesia |
| 11 | Lithuania | 36 | Iraq | 61 | Algeria | 86 | Lao PDR |
| 12 | Norway | 37 | Papua N.G. | 62 | Tanzania | 87 | Oman |
| 13 | Singapore | 38 | Croatia | 63 | Iran, I.R. | 88 | United A.E. |
| 14 | Yemen, R. | 39 | Argentina | 64 | Ireland | 89 | Bangladesh |
| 15 | Italy | 40 | Hungary | 65 | Mauritania | 90 | Vietnam |
| 16 | Madagascar | 41 | Sri Lanka | 66 | Panama | 91 | Ghana |
| 17 | New Zealand | 42 | Zambia | 67 | Tajikistan | 92 | India |
| 18 | Japan | 43 | Peru | 68 | Colombia | 93 | Qatar |
| 19 | Greece | 44 | Paraguay | 69 | Bulgaria | 94 | Nigeria |
| 20 | Denmark | 45 | Turkiye | 70 | Chile | 95 | Kazakhstan |
| 21 | Uruguay | 46 | Luxembourg | 71 | Egypt, A. R. | 96 | Cambodia |
| 22 | Germany | 47 | Brazil | 72 | Kenya | 97 | Chad |
| 23 | France | 48 | Australia | 73 | Cameroon | 98 | China |
| 24 | Netherlands | 49 | Lebanon | 74 | Pakistan | 99 | Myanmar |
| 25 | Austria | 50 | South Africa | 75 | Korea, R. | 100 | Azerbaijan |

Table S9: Ranking of countries regarding GHGs reduction with intensity average 2000-2019

| Ranking | Countries | Ranking | Countries | Ranking | Countries | Ranking | Countries |
| --- | --- | --- | --- | --- | --- | --- | --- |
| 1 | Zimbabwe | 26 | Azerbaijan | 51 | Oman | 76 | Chad |
| 2 | Denmark | 27 | Israel | 52 | South Africa | 77 | Bahrain |
| 3 | Belgium | 28 | Yemen, R. | 53 | Sudan | 78 | Romania |
| 4 | Greece | 29 | Colombia | 54 | Bangladesh | 79 | Saudi Arabia |
| 5 | Hungary | 30 | United K. | 55 | Paraguay | 80 | Turkiye |
| 6 | Italy | 31 | Indonesia | 56 | Pakistan | 81 | Kazakhstan |
| 7 | Germany | 32 | Argentina | 57 | Ghana | 82 | Lithuania |
| 8 | Ireland | 33 | Norway | 58 | Tunisia | 83 | Peru |
| 9 | France | 34 | Mexico | 59 | Ukraine | 84 | Sri Lanka |
| 10 | Netherlands | 35 | Switzerland | 60 | Morocco | 85 | Jordan |
| 11 | U.S. | 36 | Luxembourg | 61 | Cambodia | 86 | Singapore |
| 12 | Cuba | 37 | Spain | 62 | Iran, I.R. | 87 | Mali |
| 13 | Finland | 38 | Philippines | 63 | Senegal | 88 | Panama |
| 14 | Bulgaria | 39 | Korea, R. | 64 | Papua N.G. | 89 | Tanzania |
| 15 | Japan | 40 | Domin.R. | 65 | India | 90 | Vietnam |
| 16 | Australia | 41 | Sweden | 66 | Mauritania | 91 | Zambia |
| 17 | Cameroon | 42 | Iraq | 67 | Nepal | 92 | Uganda |
| 18 | Croatia | 43 | Myanmar | 68 | Egypt, A. R. | 93 | Lao PDR |
| 19 | Syrian A.R. | 44 | Brazil | 69 | Kuwait | 94 | Uzbekistan |
| 20 | Poland | 45 | Uruguay | 70 | Kyrgyz R. | 95 | Mongolia |
| 21 | Portugal | 46 | Chile | 71 | Malaysia | 96 | China |
| 22 | Austria | 47 | Bosnia H. | 72 | Kenya | 97 | Mozambique |
| 23 | Russian F. | 48 | Madagascar | 73 | Thailand | 98 | Turkmenistan |
| 24 | Nigeria | 49 | New Zealand | 74 | United A.E. | 99 | Tajikistan |
| 25 | Canada | 50 | Algeria | 75 | Lebanon | 100 | Qatar |
